# Gender and multilingual bias in observations of children with a developmental language disorder

**DOI:** 10.3389/fpsyg.2025.1572727

**Published:** 2025-04-28

**Authors:** Jitske de Vries, Max van der Velde, Bernard Veldkamp, Britt Hakvoort, Ebbo Bulder, Aafke Essen, Kim Schildkamp

**Affiliations:** ^1^ELAN Teacher Development, Faculty of Behavioural, Management and Social Sciences, University of Twente, Enschede, Netherlands; ^2^Auris, Rotterdam, Netherlands; ^3^Oberon, Utrecht, Netherlands

**Keywords:** developmental language disorder, gender, multilingualism, bias, text mining

## Abstract

**Purpose:**

The aim of our study was to clarify DLD characteristics specific to boys and girls and monolingual and multilingual children, including the detection of possible bias in observations made by speech-language therapists.

**Methods:**

We used text-mining techniques on existing individual treatment plans for children diagnosed with DLD (*N* = 994) written by speech-language therapists. Specific analyses included analyses of unigrams, bigrams, and trigrams within lines (*N* = 9,092) of individual treatment plans, followed by sentiment analyses of these unigrams, bigrams, and trigrams.

**Results:**

Not only were girls described with more negative words but the focus of the identified DLD characteristics also differed. Boys were described more in terms of tasks and girls in terms of personal characteristics, specifically hearing problems. Multilingual children were described far more negatively by their speech-language therapists than monolingual children, combined with what appeared to be a somewhat stronger focus on vocabulary in observations.

**Conclusion:**

The found differences can be due either to bias or actual differences in characteristics between these groups. Screening procedures should be adapted to detect these children earlier, and speech-language therapists should be made aware of the differences in their observations of girls and multilingual children with DLD to avoid bias.

## 1 Introduction

Language difficulty can have multiple causes, such as lack of language input from the environment or deficits in language centers in the brain. When language difficulties persist without a clear etiology, we speak of developmental language disorder (DLD). DLD is a neurobiological disorder that impairs a child’s ability to learn and use language, as expected based on age and cognitive development (Tomblin et al., 1997). These difficulties in acquiring language cannot be explained by impairments in hearing, motor skills, neurological conditions, or cognition (Bishop et al., 2017; Gerrits et al., 2017; Leonard, 2014). It is estimated that approximately 5–7% of children suffer from DLD (Gerrits and Van, 2012; Tomblin et al., 1997), while a quarter of all children struggle with a language development delay (Meelissen et al., 2023). Moreover, DLD can affect different (combinations of) language areas in each child. Typical subcategories of developmental language disorders are expressive language disorder (i.e., use of language), receptive language disorder (i.e., understanding of language), and a combination of these. Children with DLD often have late-onset babbling, which normally occurs between 6 and 8 months, and produce no single words at all, commonly occuring around 12 months (Visser-Bochane et al., 2017). Instead, these children often do not start to speak at all between 2 and 3 years of age, sometimes not even before they are 4 years old. As children with DLD grow older, they may have difficulty understanding what others say, speaking complete sentences, or finding the right words to express their thoughts. They may also have difficulty following directions or understanding abstract concepts (Glasby et al., 2022).

Examples of common problems teachers may observe in their classrooms with children with DLD include a lack of interest in verbal communication and therefore difficulty in interacting with them, which may also look like other (co-morbid) problems, such as attention deficit hyperactivity disorder or autism (e.g., Mendez-Freije et al., 2023). Children with DLD may also seem less happy, more frustrated, and lack the ability to concentrate on tasks (Coster and Goorhuis-Brouwer, 1998). Often, children with DLD show less interest in certain activities, such as pretend play, and lack interest in books and storytelling. The inability to participate in such activities may lead to social withdrawal, including limited communication and eye contact with others (Gerrits et al., 2017). Moreover, it may be more difficult to establish good contact with peers and are often more prone to being bullied (van den Bedem et al., 2020). Ultimately, children with DLD are more likely to experience negative outcomes in social, academic, and vocational domains (Conti-Ramsden et al., 2018; Nippold and Tomblin, 2014). Thus, it remains important to act on and treat DLD in a timely manner, as without this, problems in children with DLD can accumulate faster (Gerrits et al., 2017).

### 1.1 Bias in developmental language disorder

Despite the common problems that children with DLD share, their (background) characteristics may differ.

Due to these differences, it is not surprising that some bias regarding the identification of DLD exists, particularly with regard to the gender and multilingualism of children (Wiefferink et al., 2020). Being a boy is a predictor of persistent language delay and DLD (Chilosi et al., 2023). Not only are boys diagnosed approximately 10 months earlier than girls (Uilenburg et al., 2018), the ratio of diagnosed boys to girls also varies depending on the type of language disorder: 4.4:1 for isolated phonological disorders, 2.6:1 for persistent expressive disorders, and 2:1 for receptive-expressive disorders (Chilosi et al., 2023). Overall, The male-to-female prevalence ratio for DLD (Developmental Language Disorder) is estimated at 3:1 (Tomblin et al., 1997), while the ratio of receiving care is almost just as high, with 2.55:1 (Lindsay and Strand, 2016). It is not unlikely that boys and girls may show differences in social behavior that either amplify (for boys) or mask (for girls) their language challenges (McGregor, 2020). In familial settings, more male relatives are diagnosed with DLD than are female relatives (Flax et al., 2003). The fact that boys are more likely to be identified and receive clinical attention for language-related issues rather than reflecting an inherent gender difference in susceptibility to DLD is called referral bias (Nudel et al., 2023). In fact, boys and girls with DLD have comparable scores on most cognitive measures, including non-verbal IQ (Wiefferink et al., 2020). The latter is also true both before and after treatment, indicating that differentiation between boys and girls in interventions is not necessary (Vermeij et al., 2022). The fact that there is referral bias may also point to the importance of differences in behavioral characteristics between boys and girls with DLD. Although this is not always the case (Shimko et al., 2020), boys with DLD more often have co-morbid behavioral and attentional problems than girls (Bishop et al., 2017; Wiefferink et al., 2020). Examples of such problems include aggression and hyperactivity (Uilenburg et al., 2018). Girls, on the other hand, are more likely to have internalized problems such as anxiety and shyness. DLD-related problems in boys may also be more conspicuous than in girls with DLD for these reasons (Wallentin, 2020), which could also explain the presence of referral bias.

Problems with timely referral and diagnosis also exist for multilingual (in most cases, bilingual) children (Peña et al., 2011). Compared to monolingual children, their referral is often 3 months later (Wiefferink et al., 2020), despite the lack of evidence that learning multiple languages is more difficult for a child with DLD than learning only one language (Peña et al., 2011). Furthermore, scores on non-verbal tests are similar for monolingual and multilingual children, but not for verbal tests. On verbal tests, multilingual children with DLD score much lower than monolingual children (Schachinger-Lorentzon et al., 2023). The later referral of multilingual children may follow from the fact that multilingualism per definition reduces the amount of language input in a given language, as children are spoken in multiple languages. This in turn reduces the size of a child’s vocabulary per language however, the size of the overall vocabulary is increased (Boerma et al., 2017). A lack of language input, and by extension output, is also a common cause of language delay in children, which makes the identification of DLD more difficult. In addition, clinical markers for multilingual children with DLD differ for different languages, as sentence structure and grammar differ per language (Garaffa et al., 2019). Moreover, testing children using in a language that is not their own may pose problems, as vocabulary is also partially culturally influenced (Law et al., 2000). There is, however, a (small) rise in attention for alternative diagnosis of children with a multicultural background, as more tools for the diagnosis of these children are developed (Mostaert and Leysen, 2024; Pratt et al., 2022). Along with problems regarding vocabulary size, multilingual children diagnosed with DLD often have more complex types of DLD. Multilingual children with DLD generally exhibit both receptive and expressive language disorders, whereas monolingual children often only have one of these disorders. More behavioral and cognitive problems can also occur in multilingual children at the time of referral (Wiefferink et al., 2020). Even with referral, the problems of multilingual children with DLD are persistent and difficult to overcome (Schachinger-Lorentzon et al., 2023).

Early diagnosis of DLD is important, and as described above, it seems more problematic for girls and multilingual children. It is therefore useful to explore whether there are differences in how girls and multilingual children with DLD are observed by speech-language therapists compared with boys and monolingual children. Also, more commonly used l screening guides and tools, may be better aligned with the characteristics of boys and monolingual children. It is therefore even more important to understand that how characteristics of boys and girls and monolingual and multilingual children with DLD differ, so that assessment tools can be adapted accordingly or these differences can be taken into account when using them.

### 1.2 Data to help detect bias in developmental language disorder

With the increasing digitalization of education, more data are available, which can be useful for identifying indicators of bias in the diagnosis of DLD. It is especially important to recognize data patterns specific to girls and multilingual children, who currently appear to be the most subject to bias. There is a lot of data available in schools, referred to as “big data,” including data regarding characteristics that might help signal children with DLD earlier. *Big data* in education refers to the following.

A variety of data types about various levels of educational systems, complex and social interactions, stored at different places and in multiple systems, which need to be connected in order to be able to analyze processes taking place in education and to improve education (Veldkamp et al., 2021, p. 267).

As such, the use of big data involves linking previously separated data files and analyzing these linked files to find answers to specific questions and to discover unexpected correlations (Veldkamp et al., 2021). As schools increasingly digitize their educational processes, they capture more educational data in databases, including student tracking systems and online educational applications. The recent surge in remote learning owing to the COVID-19 outbreak has further emphasized the importance of collecting such data. These data can be divided into structured and unstructured categories.

Most schools in The Netherlands work with a standardized student-tracking system in which all data of individual pupils or students are saved. These include “background” data such as student ID, gender, date of birth, family situation, and contact details, as well as specific assessment information. The last includes information such as grade level, grades per subject, types of assessment administered, and whether a student has repeated a grade. In the case of DLD, this means that pupils have “extra” data, such as the results of speech-language tests and IQ tests, which are not regularly conducted in regular primary education.

Much unstructured data exist in education that are currently not often used for analysis (Veldkamp et al., 2021). For example, educational software used in many schools can log records such as the number of clicks, time spent in the environment, and answers given by the students. Reported qualitative data also fall within this category, such as observations and reflections on children’s behavior and performance. In the case of children with DLD, unstructured data can include personalized treatment plans, which include individual goal setting, as well as observations of and reflections about the child by the speech-language therapist.

### 1.3 Aim of the study

Currently, little is known about the differences in characteristics related to DLD between boys and girls, and monolingual and multilingual children. This negatively influences the timely referral and diagnosis of girls and multilingual children (Wiefferink et al., 2020). In this study, we used a rich dataset of 9,092 texts belonging to personalized treatment plans written by speech-language therapists of 994 children diagnosed with DLD in primary school groups 0–4 (comparable to kindergarten to grade 2). We linked these texts with the gender and possible multilingualism of a child, which makes it possible to identify differential DLD characteristics (or “red flags”) related to gender and multilingualism. These characteristics primarily focus on the socio-emotional aspects of DLD, such as a child’s ability to interact with peers, as this is the main content available in personalized treatment plans. Next to this data, we also include some structured data of these students, which are scores on yearly administered national assessments aimed at measuring the cognitive development of children in spelling, technical reading, comprehensive reading and arithmetic. The aim of this study was to distinguish the characteristics of (boys and) girls and (monolingual and) multilingual children with DLD, which may help health care providers and teachers identify these children earlier. Furthermore, it may provide input for screening tests, which currently lack insight into the differences in DLD in girls and multilingual children. Our research questions were as follows: (1) How do the DLD-related characteristics of boys and girls with DLD differ in personalized treatment plans? (2) How do the DLD-related characteristics of monolingual and multilingual children with DLD differ in personalized treatment plans?

## 2 Methods

### 2.1 Data

The data for this study was collected by extracting texts of personalized treatment plans of children diagnosed with severe DLD, written by speech-language therapists, from pupil following systems of several special, public, education schools in The Netherlands, all situated in the Western region of The Netherlands. To write a personalized treatment plan, which are often treated as “living documents,” each speech-language therapist used a semi-structured format, including 35 standard categories by which a speech-language therapist can describe the child. These categories are, when translated from Dutch to English, among others, “learning to learn” (*n* = 1,685), “didactical development” (*n* = 1,384), “speech” (*n* = 857), “cognitive development” (*n* = 731) and “language form” (*n* = 469). For each category, an additional classification was made to determine whether an observation fell into the promotive category (*n* = 4,723) or inhibitive category (n = 4,367) in relation to the child’s development. Our dataset consisted of 9,092 Dutch texts of 994 children, who were all diagnosed with a severe form of DLD. Specifically, the number of children and associated texts included in our study in terms of the variables of interest, gender, and multiculturalism can be found in Table 1. As can be seen in Table 1, there were more boys (67.8%) than girls (or unknown) in our dataset and more monolingual children (86.5%) than multilingual children (or unknown). The mean number of texts per child was 9.14 (*SD* = 5.85).

**Table 1:** Number of children/texts per gender and multilingualism status.

Child characteristic		Children	Texts
Gender	Male	673	6,302
	Female	290	2,683
	Unknown	31	107
Multilingualism status	Monolingual	817	7,141
	Multilingual	146	1844

The mean age of all children was 93.3 months (SD = 15.2) and ranged from 57 to 137 for girls, from 60 to 147 for boys, from 57 to 147 for monolingual children, and 62 to 132 for multilingual children. For privacy reasons, we were not allowed to collect any other background characteristics during data collection. Therefore, to be able to zoom in on gender and multilingualism, we extracted this data from the texts themselves. For gender, we did this by investigating the relative prevalence of pronouns in their texts, and by analyzing the frequency of words such as “boy” (*n* = 4,182) and “girl” (*n* = 69). With regard to the monolingualism and multilingualism of a child, we determined these based on the occurrence of words in the texts such as “multilingualism” (*n* = 33) and “bilingualism” (*n* = 3), as well as the code-word “LANGUAGE” (*n* = 208), which we used to anonymize the second language a child might speak in all texts. It is important to note that these codes are somewhat less reliable, as a child could be multilingual, even though this was not mentioned by the speech-language therapist. It was not possible to identify some children and texts as belonging to a boy or girl, which means that we did not include 31 children in the analyses regarding the first research question.

Next to unstructured data, we also obtained some structured data, which were the scores on a national assessment (i.e., cito-test) and include spelling, technical reading, comprehensive reading and arithmetic. The scores for different grades were transformed to a common scale and ranged from 0 to 304.33 (spelling), 0 to 77.25 (technical reading), 53 to 160.67 (comprehensive reading), and 9.5 to 224.29 (arithmetic). For some children we had no measurement for certain assessments, and for others we had multiple measurements. In the case of the latter, we transformed these scores to one mean score. Using regression analyses, in which we corrected for age, we found no meaningful differences between girls and boys for spelling [*β* = 0.93, *t*(666) = 0.17, *p* = 0.864], for technical reading [*β* = 1.46, *t*(676) = 1.19, *p* = 0.234], and comprehensive reading [*β* = −0.19, *t*(405) = −0.09, *p* = 0.925]. However, we found a significant difference for arithmetic between girls and boys [*β* = 15.79, *t*(678) = 5.78, *p* < 0.001]. Multilingual status, again corrected for age, not a significant predictor of spelling [*β* = 4.93, *t*(666) = 0.70, *p* = 0.486], mathematics [*β* = −1.28, *t*(678) = −0.35, *p* = 0.724] or technical reading [*β* = 0.86, *t*(676) = 0.54, *p* = 0.590]. We do, however, see a trend in comprehensive reading [*β* = −5.13, *t*(405) = −1.96, *p* = 0.051], finding higher scores for monolingual children.

### 2.2 Data extraction

This study was conducted in collaboration with, but not directly under, the formal structure of the school organization. As such, we requested and received approval from the school’s data protection officer to access and utilize student data for research purposes. Although the school organization had already requested explicit consent from parents or guardians for student participation in scientific research, additional privacy measures were required due to the study being conducted partly outside the school’s internal research framework. In accordance with data protection regulations and the guidance of the data protection officer, all data had to be fully anonymized to ensure student privacy and confidentiality. As a result, only limited background information and structured data were made available for analysis. These restrictions, while essential for safeguarding personal data, influenced the scope of the study and considered into account when interpreting the findings.

The participants’ unstructured data, the treatment plans, that we used to answer the research questions, were saved by the participating school organization in ParnasSys, a pupil tracking system used in (special) primary education in The Netherlands. Although all schools in the participating school organization used ParnasSys, some deviated from the standard format. To extract unstructured data, the texts within personalized treatment plans, from ParnasSys, we developed parsers in Python to structure data. These parsers were capable of extracting all personalized treatment plans from the school organization generated within the ParnasSys. In collaboration with the school organization, we established a procedure to anonymize the texts in personalized treatment plans, which often contain the medical information of minors. The school organization anonymized texts based on a set of rules regarding the student’s name and named-entity recognition linked with manual validation of named entities. This process removed both the name and other identifying information (such as second language or the school’s location) from the texts. Finally, we constructed a dataset saved as. csv, containing the following columns: student ID, category, prohibitive/promotive, and text.

### 2.3 Data analysis

To answer the research questions, we applied a combination of text-based machine learning analyses. Specifically, after completing data collection and preparation, we conducted n-gram and sentiment analyses to identify differences in DLD-related characteristics between boys and girls, and between monolingual and multilingual children.

#### 2.3.1 Data preparation

We began data preparation by loading. Csv file into R. The tidytext (Silge and Robinson, 2016) and stopword (Mäkelä, 2020) packages were used to tokenize the texts (i.e., divide the words into, for example, single words) and remove stopwords such as articles, which are considered less useful for interpretation. We also removed some custom stop words, such as “NAME,” “PERSON, and “EVENT.” Then, all punctuation marks and numbers were removed from the texts. As described above, we added gender and multilinguistic variables using tags (or codes).

#### 2.3.2 Analysis of n-grams

N-grams are contiguous sequences of 𝑛 items (words, characters, or tokens) from a given text or speech. Unigrams refers to a single word, bigrams to two pairs of words, and trigrams to three consecutive words. Prior to starting the analysis of n-grams, we replaced any words such as “girl” or “boy” for the word “child,” and deleted words such as “multilingualism” and “bilingualism” for language status. If these defining words were not removed, they would have dominated the n-grams, resulting in their frequent occurrence and making the log odds for the n-grams less meaningful. This resulted in 6 different datasets, which were used for the analysis of n-grams and sentiment analyses: gender – unigram, gender – bigram, gender – trigram, multilingualism – unigram, multilingualism – bigram, multilingualism – trigram. We calculated the frequency of the unigrams, bigrams and trigrams using the tidyverse (Wickham et al., 2019) and tidytext (Silge and Robinson, 2016) packages. Next, weighted log odds were used to calculate the odds ratio of the distribution of features within a specific set as compared to all other sets. We used the tidylo package (Silge, 2020) to do this. To illustrate the results, we used graphs generated in Microsoft *Excel*, in which we included the 15 highest log odds per variable, and in which we manually translated the terms (unigrams, bigrams, trigrams) into English. This is why we sometimes present, for example, bigrams as unigrams, because a single word in Dutch can sometimes translate into multiple words in English. Also, sometimes n-grams appear non-sensical, as stopwords have been deleted from the full text.

#### 2.3.3 Sentiment analysis

Sentiment analysis is used to measure the sentiment of a given piece of text, usually to determine whether it has a positive or negative connotation (Zhou and Ye, 2023). Sentiment analyses were conducted to detect sentiments expressed in unigrams, bigrams, and trigrams using the tidyverse (Wickham et al., 2019), tidytext (Silge and Robinson, 2016), and xml2 (Allaire and Grolemund, 2021) packages, and the EmoLex dictionary (Mohammad and Turney, 2013). The EmoLex dictionary contained 10 sentiments: positive, negative, fear, sadness, anger, disgust, surprise, anticipation, trust, and joy, and was used to give each word a score ranging between 0 and 1. The extracted sentiments were used to conduct logistic regression analyses to investigate whether sentiment scores could be used to predict whether a text referred to a boy/girl or a monolingual/multilingual child.

## 3 Results

### 3.1 Gender differences in DLD

#### 3.1.1 Analysis of unigrams, bigrams, and trigrams

**Figure 1:**
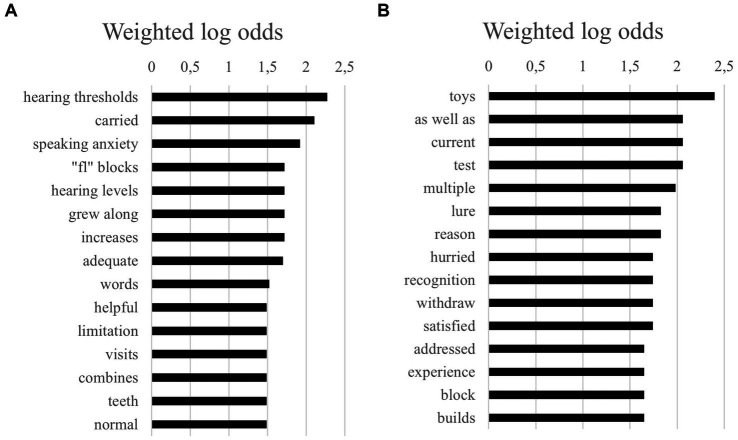
**(A)** The 15 highest log odds of unigrams for girls. **(B)** The 15 highest log odds of unigrams for boys.

Below, we present the 15 highest log odds of unigrams (i.e., single words) by gender (Figures 1A,B). The unigrams show that, in general, few differences of interest were present between boys and girls. However, one remarkable finding concerned the prevalence of mentions of “hearing thresholds” (combined with “hearing levels”) and “speaking anxiety,” which were far more frequent in texts about girls compared to boys. Specifically, for example, the chances of a girl being described as having a fear of speaking were estimated to be 6.69 times higher than those for boys.

**Figure 2:**
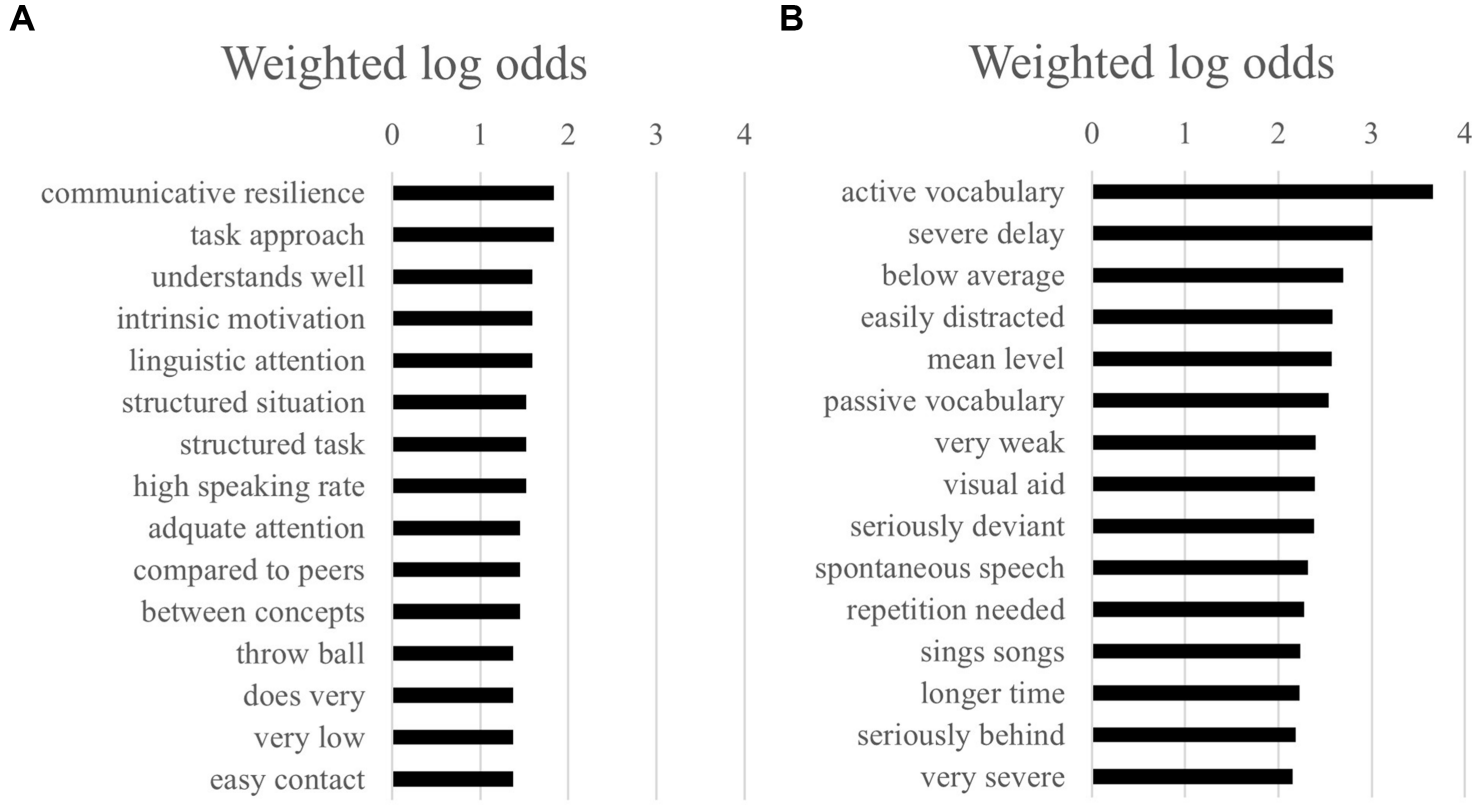
**(A)** The 15 highest log odds of bigrams for girls. **(B)** The 15 highest log odds of bigrams for boys.

However, as shown in Figures 2A,B, differences were more frequent when comparing bigrams (i.e., combinations of two words) between boys and girls. It was noticeable that girls had more negative bigrams, such as “severe delay” and “seriously deviant,” compared to boys. Based on the 15 highest log odds of bigrams shown in Figures 2A,B, the combined log odds of girls being more negatively described (in bigrams) than boys was very high at 14.8. With boys, the bigrams seemed to be more neutral, with a focus more often on task approach (e.g., “task approach,” “structured tasks,” “longer time,” “adequate attention”).

**Figure 3:**
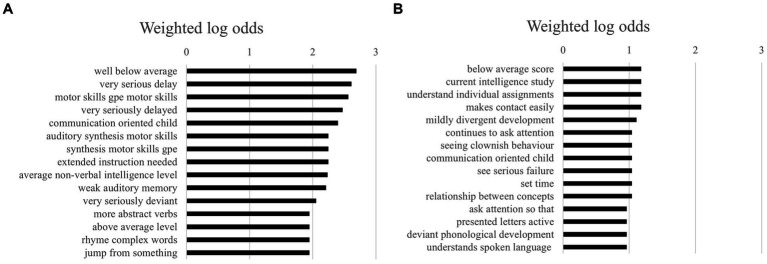
**(A)** The 15 highest log odds of trigrams for girls. **(B)** The 15 highest log odds of trigrams for boys.

For trigrams (i.e., combinations of three words), as shown in Figures 3A,B, we found that girls were described even more negatively (such as “well below average,” “very seriously deviant,” “and “very seriously delayed”). Boys, on the other hand, were, again, generally described in neutral terms, stressing external behaviors such as asking for attention, showing clownish behavior, and making contact easily.

#### 3.1.2 Sentiment analysis

Next, we analyzed the sentiments (or tone of the words) that occurred in the texts referring to boys and girls for all unigrams, bigrams, and trigrams. Based on the mean scores, we identified differences in the tone speech-language therapists used when writing about boys or girls. See Table 2 for the sentiment scores for each gender.

**Table 2:** Mean and standard deviation per sentiment score for boys and girls.

N-grams	Gender	Anger	Anticipation	Disgust	Fear	Joy	Negative	Positive	Sad	Surprise	Trust
Uni	Girl	5.79 (5.45)	7.87 (6.84)	1.73 (2.42)	4.91 (4.97)	6.00 (5.91)	19.33 (16.17)	21.40 (17.82)	7.58 (6.74)	4.32 (4.21)	9.94 (8.22)
	Boy	5.93 (5.38)	8.51 (7.69)	1.87 (2.54)	5.03 (4.75)	6.79 (6.81)	20.4 (16.72)	24.0 (21.57)	8.03 (7.07)	4.83 (5.65)	10.8 (9.93)
Bi	Girl	4.51 (4.54)	6.65 (6.17)	1.27 (2.03)	3.10 (3.59)	4.88 (5.29)	15.9 (13.78)	16.4 (14.01)	6.15 (5.96)	3.83 (3.84)	7.76 (6.82)
	Boy	4.41 (4.53)	7.14 (6.60)	1.38 (2.17)	3.09 (3.62)	5.54 (5.78)	16.2 (14.40)	18.3 (17.30)	6.15 (6.07)	4.27 (5.10)	8.18 (7.85)
Tri	Girl	2.45 (3.05)	3.39 (3.66)	0.71 (1.44)	1.57 (2.23)	2.45 (3.18)	9.45 (9.37)	8.31 (8.06)	3.85 (5.56)	1.92 (2.41)	4.11 (4.26)
	Boy	2.53 (3.49)	3.60 (3.91)	0.81 (1.76)	1.71 (2.72)	2.78 (3.45)	9.65 (10.29)	9.61 (10.39)	3.74 (4.55)	2.21 (3.14)	4.35 (4.90)

It was noticeable that texts about girls were generally less positive than those about boys, even though they were not more negative (i.e., neutral). This is contrary to what we found with our n-grams, where girls were described with more negative words than boys were. Generally, we found a correlation between the degree of positivity and negativity of the texts; when a child was described more positively, the child was also described more negatively (*r* = 0.82, *p* < 0.001). This may be explained by the fact that several speech-language therapists are usually involved in writing personalized treatment plans, and some speech-language therapists may be used to writing using more extreme language. To a lesser extent, this was also true for bigrams (*r* = 0.75, *p* < 0.001) and trigrams (*r* = 0.67, *p* < 0.001). We also investigated whether any of the sentiments could be used as a predictor of gender, first generally for the positive or negative tone of the text and second for positive and negative sentiments separately. The chances were higher for a positive bigram (*OR* = 0.985, *p* = 0.05, 95% CI [0.971; 0.998]) and trigram (*OR* = 0.975, *p* = 0.05, 95% CI [0.955; 0.966]) for a boy than for a girl. Further sentiment analyses showed that for bigrams, the odds of having a higher trust score were higher for boys than for girls, but only very slightly (*OR* = 1.006, *p* = 0.05, 95% CI [1.054; 1.111]).

### 3.2 Multilingualism differences in DLD

#### 3.2.1 Analysis of unigrams, bigrams, and trigrams

**Figure 4:**
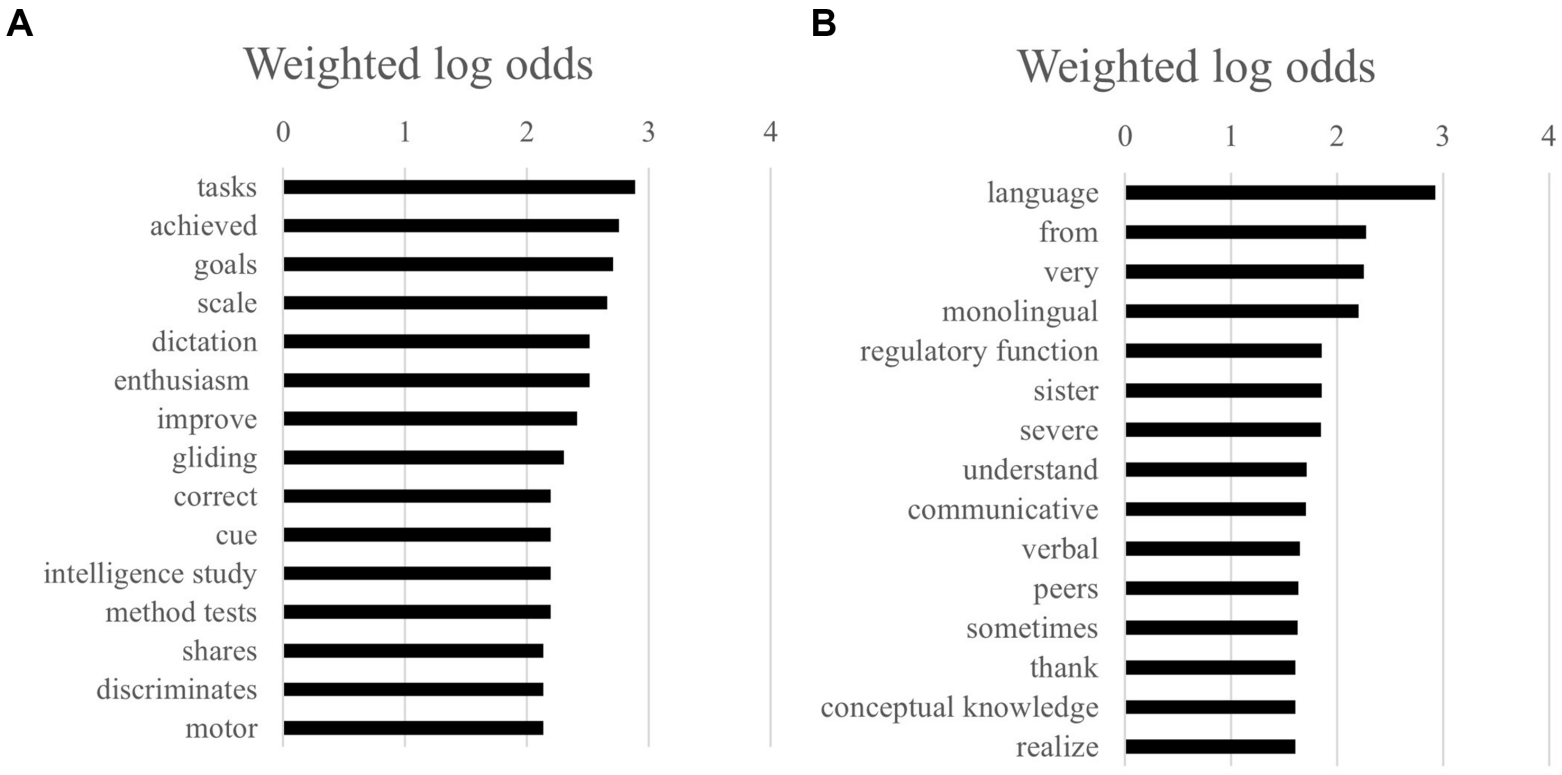
**(A)** The 15 highest log odds of unigrams for monolingual children. **(B)** The 15 highest log odds of unigrams for multilingual children.

The top 15 unigrams for monolingual and multilingual children showed a few interesting differences (see Figures 4A,B). For monolingual children, the focus was seemingly stronger on diagnosis of DLD itself (i.e., “tasks,” “method test,” “intelligence study” compared to multilingual children). On the other hand, bigrams for multilingual children were more general, such as “language,” “understand,” and “communicative.”

**Figure 5:**
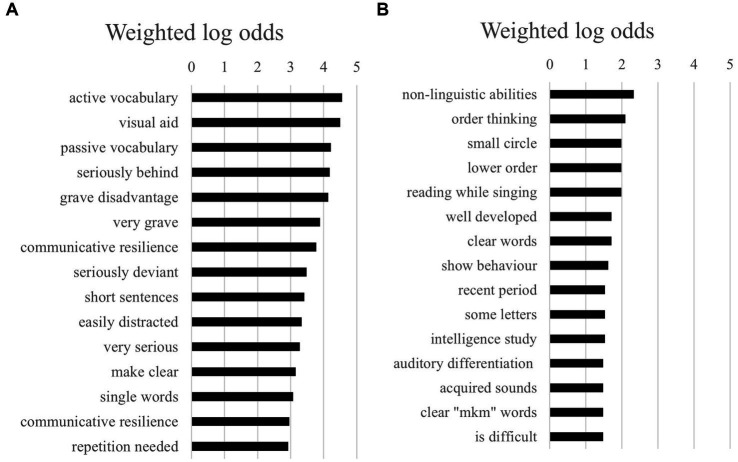
**(A)** The 15 highest log odds of bigrams for monolingual children. **(B)** The 15 highest log odds of bigrams for multilingual children.

For bigrams, more interesting differences existed between texts of monolingual and multilingual children (see Figures 5A,B). It is especially noteworthy that multilingual children were more often described with negative words such as “serious disadvantage” and “seriously behind” seriously behind’ than monolingual children. Vocabulary, both active and passive, also seemed to be a larger theme in texts of multilingual children than monolingual children. In the case of monolingual children, the bigrams in the texts were more often about instruction (e.g., “extended instruction”) and about language domains (e.g., “linguistic capabilities,” “clear words”).

**Figure 6:**
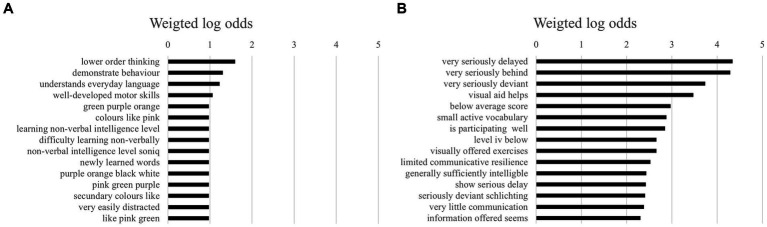
**(A)** The 15 highest log odds of trigrams for multilingual children. **(B)** The 15 highest log odds of trigrams for multilingual children.

For trigrams, multilingual children were described far more negatively than monolingual children (Figures 6A,B). The top three bigrams for multilingual children were “very seriously delayed,” “very seriously behind,” and “very seriously deviant.” The combined log odds of being negatively described by these three bigrams as multilingual (compared to monolingual) was 12.36, which is very high.

#### 3.2.2 Sentiment analysis

Next, we analyzed whether the same sentiments occurred in monolingual and multilingual children. Based on the mean scores, we identified differences in all sentiments of speech-language therapists when writing about monolingual or multilingual children. Whether the sentiment of a text was negative or positive differed, especially between monolingual and multilingual children (Table 3).

**Table 3:** Mean and standard deviation per sentiment score for monolingual and multilingual children.

N-grams	Multilingual status	Anger	Anticipation	Disgust	Fear	Joy	Negative	Positive	Sad	Surprise	Trust
Uni	Mono	5.40 (5.25)	7.66 (7.10)	1.64 (2.34)	4.57 (4.62)	5.99 (6.21)	18.23 (15.74)	21.14 (19.33)	7.09 (6.56)	4.36 (5.03)	9.59 (9.07)
	Multi	7.60 (5.73)	10.49 (8.67)	2.55 (3.06)	6.49 (5.47)	8.49 (7.76)	27.07 (18.65)	30.70 (24.62)	11.23 (7.98)	5.54 (6.12)	13.97 (10.56)
Bi	Mono	4.00 (4.29)	6.46 (6.10)	1.16 (1.86)	2.78 (3.36)	4.92 (5.25)	14.42 (13.26)	16.18 (15.41)	5.45 (5.63)	3.87 (4.49)	7.35 (7.11)
	Multi	6.17 (5.21)	8.76 (7.85)	2.17 (3.03)	4.35 (4.46)	6.72 (7.12)	23.11 (16.61)	23.32 (19.77)	9.37 (7.01)	4.88 (5.76)	10.52 (9.06)
Tri	Mono	2.25 (3.17)	3.24 (3.50)	0.64 (1.33)	1.49 (2.35)	2.43 (3.06)	8.54 (9.09)	8.29 (8.87)	3.29 (4.13)	1.97 (2.74)	3.86 (4.32)
	Multi	3.61 (3.96)	4.69 (5.01)	1.47 (2.80)	2.44 (3.47)	3.70 (4.46)	14.41 (12.80)	13.04 (12.58)	6.15 (5.84)	2.69 (3.68)	5.93 (6.03)

In general, when unigrams (*r* = 0.83, *p* < 0.001), bigrams (*r* = 0.76, *p* < 0.001) and trigrams (*r* = 0.67, *p* < 0.001) referring to a child were more strongly positive in tone, they were also more strongly negative in tone. Generally, it seemed that multilingual children were described more expressively (with more extreme levels of sentiment), that is, more negative and positive. This was also true for the other sentiments investigated. In further regression analyses, we found that sentiments in a text could also predict whether the text belonged to a monolingual or multilingual child. First, we ran regression analyses separately for the negativeness or positivity of unigrams, bigrams, and trigrams. In the case of unigrams (*OR* = 1.024, *p* = 0.01, 95% CI [1.007; 1.041]), bigrams (*OR* = 1.042, *p* = 0.001, 95% CI [1.023; 1.059]), and trigrams (*OR* = 1.013, *p* = 0.001, 95% CI [1.018; 1.061]), the odds of belonging to a multilingual child were slightly higher if their tone was negative. Second, when further investigating other sentiments in the texts, we also found differing odds for monolingual and multilingual children regarding other sentiments within the unigrams, bigrams, and trigrams. For unigrams, the chances of greater sadness (*OR* = 1.126, *p* = 0.05, 95% CI [1.041; 1.217]) and less surprise (*OR* = 0.894, *p* = 0.05, 95% CI [0.830; 0.963]) were higher for multilingual children. For bigrams and trigrams, very similar odds ratios were found for sadness and surprise. In addition, the chances of detecting a disgusted tone in the observations of multilingual children were slightly higher in the bigrams (*OR* = 1.144, *p* = 0.05, 95% CI [1.030; 1.272]) and trigrams (*OR* = 1.177, *p* = 0.05, 95% CI [1.040; 1.331]), with a lower chance of detecting fear in the observations of multilingual children in both bigrams (*OR* = 0.905, *p* = 0.05, 95% CI [0.832; 0.985]) and trigrams (*OR* = 0.886, *p* = 0.05, 95% CI [0.799; 0.981]).

## 4 Discussion

The aim of our study was to clarify DLD-related characteristics for boys and girls and monolingual and multilingual children, specifically looking at bias, using text-mining techniques on individual treatment plans written by speech-language therapists. This study confirms that bias is likely to be a problem in the timely diagnosis of DLD in girls and multilingual children.

### 4.1 Boys and girls

For boys and girls, we found characteristics that are largely in line with the current assumptions of speech-language therapists, as girls showed more internalizing behavior and boys showed more externalized behavior (Uilenburg et al., 2018). For example, one of the top terms used for girls was “fear of speaking,” which could be either an indicator of difficulties with expression, a characteristic commonly strongly related to DLD. In addition, girls with DLD are more likely to have internalized behavior tendencies (e.g., shyness, introversion, or emotional distress), which can lead to social anxiety or general communication apprehension. The topics of texts referring to girls contained many more negative words, such as “well below average” and “very seriously delayed,” possibly suggesting that the girls in our dataset had a more severe form of DLD than the boys in our dataset. This was also in line with the sentiments we found in unigrams, bigrams, and trigrams. Overall, texts referring to boys had a more positive tone.

Whether these results reflect real differences in characteristics between boys and girls is verifiable based on our data as we do know that girls and boys and monolingual children and multilingual children did mostly score similarly on the yearly-administered national assessments. We found that boys scored significantly higher on arithmetic and monolingual children on comprehensive reading, however, this does not explain the magnitude of negative words we found describing girls and multilingual children. It could be that the fact that girls showed DLD-related signs that were described as far more serious and deviant compared to boys was due to the fact that internalized behavior has to be worse before it can be detected as a signal for DLD. Additionally, this could have been influenced by (referral) bias. While boys are more frequently referred for DLD diagnosis than girls, no significant difference in the severity of symptoms is expected between boys and girls (Vermeij et al., 2022). All children with DLD, girls and boys, in our dataset have been diagnosed with a severe form of DLD, although we do not know the specific type of DLD. That our findings point toward bias is in line with multiple studies (Calder et al., 2022; Wiefferink et al., 2020), which have shown that there are concerns regarding bias. Another indicator of bias is the fact that our sample of children with DLD had a large overrepresentation of boys compared to girls, which is similar to findings in prevalence studies (Lindsay and Strand, 2016; Tomblin et al., 1997). This is, as mentioned above,despite the fact that there is real doubt whether DLD should occur more frequently in boys than in girls (Calder et al., 2022; Vermeij et al., 2022).

Other notable characteristics were “hearing levels” and “hearing thresholds” for girls. This could be because this characteristic is more often a co-morbid problem in girls. As girls generally show more internalized behavior, speech-language therapists may doubt whether language problems are due to hearing or are an actual symptom of a girl with DLD. The treatment of children with DLD may differ. If a language delay is only the result of a hearing deficiency, one might expect that language difficulties in children with cochlear implants or hearing aids would decrease because of auditory support. However, this is not the case for children with both DLD and hearing impairments.

For boys, it seems that the texts more often discussed tasks compared to those for girls, which has not been shown before in research on DLD in young children. It might be that how a boy with DLD approaches a task is more conspicuous than that of girls, with a greater need to structure the task well. In addition, the (positive) work attitude of boys was described more often by speech-language therapists, showing a stronger focus on behavior in boys with DLD than in girls with DLD, as suggested by other research findings (Calder et al., 2022). This is in line with the finding that boys with DLD are more often referred compared to girls, as mentioned above, as they may be more easily noticed (McGregor, 2020).

### 4.2 Monolingual and multilingual children

For monolingual and multilingual children, a similar pattern was found regarding the severity of the language used, specifically for multilingual children. The explanation for this finding could be similar to that of the findings regarding girls: the severity of the DLD has to be higher in order to be detected, as there are distracting co-morbid problems, such as shyness, quietness, and more internalized behavior in general in children who speak a language less well compared to their peers (Wiefferink et al., 2020). Moreover, multilingual children diagnosed with DLD often both expressive and receptive problems, amplified by a language delay, which possibly can account for the more frequent use of negative words, such as “severity.” It is likely that only complex cases are diagnosed, and more “typical” cases of DLD in multilingual children are not identified at all (Wiefferink et al., 2020). Multilingualism often masks DLD, and it often co-occurs with non-native speaking parents, which may find it more difficult to find support for their child with potentially severe language problems (Thomas et al., 2019). However, it should be noted that all children in our dataset were diagnosed with a severe form of DLD, including none with mild cases of DLD.

It is also unsurprising that speech-language therapists are more strongly focused on active and passive vocabulary, as shown by the bigrams in the texts referring to these multilingual children, as this can play a role in the development of children with DLD develop (Boerma et al., 2017). Generally, multilingual children had more bigrams related to language (and the severity of their backlog in this regard), whereas monolingual children had topics with a more even distribution over all domains related to DLD, which was not limited to receptive and perceptive issues but also included behavioral problems and attention deficits. Finally, as expected, monolingual children were described with unigrams, such as “gliding” and “method tests” (translated from “methodetoets” in Dutch”), which are more commonly used as early signals for DLD (Dutch Association for Speech Therapy and Phoniatrics, 2017).

### 4.3 Limitations

There are some limitations regarding the data that we have used for this study. Constrained by the requirement to ensure the anonymity of the students, we were unable to link the data of children to other relevant data, such as family situation, IQ, and other relevant measures. The fact that we had to infer the gender and multilinguistic status of children from the texts also amounts to some uncertainty in the presented results. Especially in the case of multilingual status, it may be the case that some multilingual children have not been categorized as such, which, in turn, can have narrowed the results for multilingual children.

Much larger, currently non-existent, datasets are required when seeking to combine richer sets of data for children, while simultaneously ensuring the anonymity of these children. In addition, further research is needed to validate our findings and to establish that our findings can indeed be attributed to bias. In line with this, we were not able to investigate whether any interaction effects existed between gender and multilingualism, specifically in multilingual girls, as we did not have sufficient data available. This also limited our ability to investigate how different subtypes of DLD, such as expressive, receptive, or a combination of these, are described by speech-language therapists.

Additionally, the results of the sentiment analyses were not completely in line with the findings regarding differences in wording (e.g., more negative for girls/multilingual children). Thus, sentiment analyses might need further refinement to be able to be used on this specific type of data. A different type of dictionary might help to find interesting differences in different types of children with DLD. The sentiments expressed by speech-language therapists in texts about boys and girls and monolingual and multilingual children might further explain why differences in DLD characteristics were observed.

### 4.4 Recommendations

The results of this study could influence the weight assigned to certain characteristics when attempting to diagnose DLD, for example, during screening procedures. Given the previous research on referral bias (Wiefferink et al., 2020) and the observed differences in descriptions in the personalized treatment plans of speech-language therapists, it is possible that double standards are being applied. This could negatively affect the timely diagnosis and treatment of girls and multilingual children. More attention, for example, to issues related to hearing, could be given to girls with DLD, as they seemingly have more hearing problems than boys.

In line with earlier research, only complex cases of DLD for children with multilingualism were detected, whereas less complex cases often went unnoticed and untreated (Wiefferink et al., 2020). There is a need to increase awareness regarding the unequal distribution of these characteristics to treat possible (referral) bias, specifically for speech-language therapists who provide diagnoses and treatments for children with (possible) DLD. Already suggestions to better detect DLD in multilingual children exist, which includes focusing on language experience, the length of exposure to the second language, the linguistic characteristics of the child’s first language and the specific clinical markers of DLD in all languages. A good clinical marker for multilingual children with DLD is, for example, difficulty with repeating sentences (Schwob et al., 2021). It is important to help speech-language therapists to understand and use these diagnostic tools, as it can otherwise not be expected of them to signal DLD in languages strange for them. An example of how this can be done is illustrated in the case study by Hamdani et al. (2024).

For future research, it would also be of interest to investigate whether there are any interaction effects between gender and multilingual status, especially for multilingual girls since this group is marginalized more often. For this group, but also generally for girls and multilingual children, we do not know what the specific effects of referral bias are. To investigate this, longitudinal research would be beneficial.

## Data Availability

The data analyzed in this study is subject to the following licenses/restrictions: the dataset is completely anonymized, as it contains sensitive information of children, and cannot be shared with third parties beyond this research project. Requests to access these datasets should be directed to JV, j.a.devries@utwente.nl.
